# Auriculotherapy to control chemotherapy-induced nausea and vomiting in patients with cancer: protocol of a systematic review

**DOI:** 10.1186/s13643-019-1124-3

**Published:** 2019-08-15

**Authors:** Ruan Nilton Rodrigues Melo, Stephanie Carolina Francisco, Caroline de Castro Moura, Kirsty Loudon, Namie Okino Sawada, Érika de Cássia Lopes Chaves, Tânia Couto Machado Chianca, Denismar Alves Nogueira, Si Jia Zhu, Ana Cláudia Mesquita Garcia

**Affiliations:** 1 School of Nursing, Federal University of Alfenas, Gabriel Monteiro da Silva street, 700-Centro, Alfenas, MG 30.130-100 Brazil; 2 School of Nursing, Federal University of Minas Gerais, Prof. Alfredo Balena avenue 190-Santa Efigênia, Belo Horizonte, MG 37.130-001 Brazil; 3 Edinburgh, Scotland; 4 Ribeirão Preto College of Nursing, University of São Paulo, Bandeirantes avenue 3900–University Campus-Monte Alegre, Ribeirão Preto, SP 14.040-900 Brazil; 5 Institute of Exact Sciences, Federal University of Alfenas, Gabriel Monteiro da Silva street 700-Centro, Alfenas, MG 37.130-001 Brazil; 6 Centre of Evidence-Based Medicine, Beijing University of Chinese Medicine (BUCM)–China, East road 11 of North Third Ring road-Chaoyang district, CEP(Zip code), Beijing, 1000029 China

**Keywords:** Nausea, Vomiting, Drug therapy, Neoplasms, Auricular acupuncture, Systematic review

## Abstract

**Background:**

Due to the worldwide rise in cancer incidence, and therefore the rise in the need for antineoplastic chemotherapy, it is important for both healthcare professionals and patients alike that the side effects of chemotherapy, such as chemotherapy-induced nausea and vomiting (CINV), are treated and prevented. Auriculotherapy is a type of acupuncture and may be a low-cost and safe antiemetic measure to control the side effects of chemotherapy. The goal of this systematic review is to synthesize the available evidence in the literature regarding the auriculotherapy effects to treat CINV in people with cancer.

**Methods:**

The review will only include randomized controlled trials (RCTs) that compare the clinical effects of the auriculotherapy intervention (used alone or as an add-on), with sham auriculotherapy, routine treatment with antiemetic drugs, or other non-pharmacological interventions in patients with cancer with CINV who are undergoing chemotherapy. The outcomes to be evaluated are nausea and vomiting: in acute, delayed, or anticipated stages, when induced by chemotherapy. A comprehensive search for studies will be carried out in these databases: MEDLINE via PubMed, EMBASE, CINAHL, Cochrane Central Register of Controlled Trials, ICTRP, LILACS, CUMED, IBECS, BVS MTCI Americas, Web of Science, Scopus, PEDro, CNKI, and CBMdisc up until December 31, 2018. Only articles in English, Portuguese, and Spanish will be selected. Two independent reviewers will evaluate full texts, extract data, and assess the risk of bias of eligible articles. The quality of evidence will be assessed using Grading of Recommendations, Assessment, Development and Evaluation (GRADE). A meta-analysis will be undertaken to assess the interventions and outcomes’ homogeneity, assessing statistical heterogeneity using the Cochran’s *Q* test and quantified using Higgins’ inconsistency index. If there is insufficient data for a meta-analysis, a narrative synthesis will be presented. This protocol has been prepared according to the Preferred Reporting Items for Systematic Review and Meta-Analysis Protocols (PRISMA-P) guidelines.

**Discussion:**

The results of this systematic review will summarize the strength of evidence for the use of auriculotherapy in the control of CINV of patients with cancer and will be used to identify evidence gaps.

**Systematic review registration:**

PROSPERO CRD42018117513

**Electronic supplementary material:**

The online version of this article (10.1186/s13643-019-1124-3) contains supplementary material, which is available to authorized users.

## Background

Nausea and vomiting are among the toxic side effects most frequently associated with antineoplastic chemotherapy, having a devastating effect on the quality of life of cancer patients [1, 2]. Although nausea and vomiting can result from surgery and radiotherapy, chemotherapy-induced nausea and vomiting (CINV) is potentially the most serious and distressing [1]. CINV can be classified as acute (occurs minutes or hours after the chemotherapeutic treatment and generally disappears within 24 h), delayed (occurs at least 1 day after chemotherapy and can last five or more days) and anticipated (occurs before chemotherapy and is a conditioned response to previous CINV episodes) [3]. When left untreated, CINV can affect 60 to 80% of cancer patients [4]. Moreover, this condition can undermine the antineoplastic treatment results, including, in some cases, the need to discontinue chemotherapy [5–8].

Vomiting is an instinctive defensive reaction caused by the somatoautonomic nervous reflex, which is integrated into the bulb of the encephalic trunk (brainstem). The vomiting caused by cytotoxic drugs is associated with an increase in the concentration of 5-hydroxytryptamine (5-HT) in the intestine and in the encephalic trunk (brainstem). It is believed that cytotoxic drugs cause the release of 5-HT in enterochromaffin cells in the intestinal mucosal barrier and that the 5-HT that is released stimulates the 5-HT receptors in the adjacent vagal afferent nerves. The depolarization of vagal afferent nerves stimulates the center of vomiting in the encephalic trunk (brainstem) and, eventually, causes a vomiting reflex [9]. The medications normally used to control the CINV include serotonin (5-HT_3_) receptor antagonists (RAs), neurokinin 1 (NK-1) RAs, corticosteroids, and metoclopramide, among others [10–12]. There is evidence that after prophylaxis with these antiemetic medications, the effects of acute and delayed nausea and vomiting can be avoided in up to 50% of the cases [13]. However, the high cost of these agents and their side effects, such as extra pyramidal disorders, hypotension, headache, constipation, fatigue, mouth dryness, vertigo, diarrhea, and irritability, have limited the use of these medications [14–16].

Given the growing preferences for complementary and alternative therapies (CAT), patients are interested in alternatives to standard pharmacological treatments to CINV, or even associating the use of these therapies to traditional allopathic interventions [17, 18]. The CAT generally do not require a medical prescription, they can be accessed in the community and have gained more attention in the scientific community recently [18]. According to one of the evidence directives for the practice provided by the Oncology Nursing Society, the stimulation of acupuncture points was considered a promising intervention for CINV handling [13]. One of the fields of acupuncture is auriculotherapy, which has been used for approximately 2500 years, with the goals of diagnosis and treatment of physical and psychosomatic disorders [19]. Auriculotherapy is one of the non-pharmacological methods that has been used to control CINV [10]. This intervention must be performed by qualified healthcare professionals. In Brazil, for instance, the Ministry of Health, by means of the *Coordenação Nacional de Práticas Integrativas e Complementares em Saúde* (National Coordination of Integrative and Complementary Healthcare Practices) offers a free course for “Auriculotherapy qualification for healthcare professionals of the basic attention sector,” with the objective of qualifying these professionals so they can work in the Primary Healthcare Attention sector.

The technique consists of stimulating specific points in the auricular pavilion, which is considered one of the main microsystems of the human [20, 21]. This therapy seeks to harmonize the functions of organs, viscera, and physical and mental infirmities, from the reflex that the stimulus causes in the points of the central nervous system, by means of finger acupressure, laser, electricity, different types of needles, magnetic balls, and seeds [21, 22]. In China, the birthplace of acupuncture, the existing correlation between the ear and the body was described in 500 A.C. in the classical text *Huang Di Nei Jing*, showing that all the body meridians converged towards the ear [21, 23, 24]. A modern form of the technique was developed by the end of 1950, by the French physician Paul Nogier, who created the inverted fetus map, in a way that the auricular points are displayed in this configuration [21, 24]. According to the principles of the Traditional Chinese Medicine, this therapy can restore and raise the flowing cycle *Qi* (vital energy) and *Xue* (blood) and, consequently, harmonize the functions of all living organs and tissues [25, 26]. In the Western perspective, the effects of this intervention are related to the nervous, neuroendocrine, and immune systems, in order to promote relief of the signs and symptoms of different illnesses, on top of possessing preventive aspects [19, 27]. Furthermore, the effects of auriculotherapy in the organism can be explained by neurophysiology and by reflexology [21]. The four mixed cranial nerves give the ear more than half of its innervation through the trigeminal, facial, glossopharyngeal, vagus, and cervical plexus nerves (C2/C3). This nervous composition means that the ear is a unique neurovascular organ [28]. There is evidence that suggests that areas of the encephalic trunk (brainstem) can be influenced by stimulating the ear [29].

The available literature concerning the therapeutic effects of auricular stimulation in symptoms of many disorders has been steadily growing [29]. In the case of surgery patients, there is evidence that suggests that auriculotherapy is a viable intervention for the treatment of postoperative nausea and vomiting, due to the safety, cost, and patient satisfaction [30]. Recently, a systematic review was carried out to evaluate the effectiveness of acupressure in CINV control, and according to the results, acupressure has a protective effect for this condition [31]; however, this review did not include studies with auriculotherapy. In a clinical test that determined the auricular acupressure effect in the CINV relief among women with breast cancer, the researchers concluded that the use of this intervention, along with other therapies, can alleviate CINV, without side effects [10].

As auriculotherapy is being considered a potential treatment to control CINV but there is no literature review of the available evidence regarding the effects, harms, and benefits of this intervention, the objectives of this review are as follows:

1. To synthesize the available evidence in the literature regarding the auriculotherapy effects in the CINV treatment of cancer patients.

2. To develop a clinical protocol that guides the use of auriculotherapy as a CINV treatment for cancer patients.

3. To identify possible adverse effects originated from the uses of auriculotherapy as a CINV treatment for cancer patients.

4. To suggest a core outcome sets (COS) based on the treatment of CINV using auriculotherapy.

## Methods

### Protocol and registration

This protocol is registered in the Prospective Register of Ongoing Systematic Reviews (PROSPERO) CRD42018117513 and was structured according to the Preferred Reporting Items for Systematic Review guidelines and Meta-Analysis Protocols (PRISMA-P 2015) [32–34]. The Preferred Reporting Items for Systematic Review (PRISMA-2009) will be used to report the study in the future. The PRISMA-P filled out checklist is included as an additional file (consult Additional file 1).

### Development of the research question

The research questions were developed based on the PICO (Population, Intervention, Comparator, Outcomes) strategy (Table 1) [35].

**Table 1:** Research question development according to the PICO strategy

PICO	Components
Review question	What is the auriculotherapy effect in controlling the nausea and vomiting induced by chemotherapy in patients with cancer in comparison to the Sham auriculotherapy, to the routine treatment, or other non-pharmacological interventions?
Population	Patients with cancer undergoing chemotherapeutic antineoplastic treatment
Intervention	Auriculotherapy with seeds (mustard/Vaccaria), metallic points, magnetic points, plastic points (crystals) needles (semi-permanent and systemic), *stiper*, radionic crystals, electroacupuncture, chromotherapy, laser therapy, and electromagnetic stimulation (*hai hua*)
Comparator	Sham auriculotherapy, routine treatment with antiemetic drugs, and other non-pharmacological interventions
Outcomes	Chemotherapy-induced nausea and vomiting control (acute, delayed, anticipated) and adverse events originated from the uses of auriculotherapy as a CINV treatment for patients with cancer

#### Eligibility criteria

##### Types of studies

Only randomized controlled trials of auriculotherapy to treat chemotherapy-induced nausea and vomiting in patients with cancer will be included.

##### Types of participants

Patients diagnosed with cancer, regardless of the type of tumor and staging, undergoing antineoplastic chemotherapeutic treatment, including those in palliative care, experiencing nausea and vomiting as adverse effects induced by the treatment. There will be no limit for age, gender, and ethnic origin.

##### Types of interventions/exposure and comparison

- The studies present auriculotherapy as an intervention with any kind of device (mustard or Vaccaria seeds, metallic points, magnetic points, plastic points, semi-permanent and systemic needles, *stiper*, radionic crystals, electroacupuncture, chromotherapy, laser therapy, electromagnetic stimulation [*hai hua*]). The intervention may be used by itself or in combination with other methods. The control interventions will include (a) Sham auriculotherapy (superficial needling, needling in non-points, non-penetrating sham needles, sham interventions without needling, minimal acupuncture, and needling in real points that are irrelevant) [36], (b) conventional routine treatment with antiemetic drugs, and (c) other non-pharmacological interventions.

##### Types of outcomes

The primary outcomes to be evaluated are nausea and vomiting: acute, delayed, or anticipated, induced by chemotherapy. According to a review, 25 tools were used to evaluate nausea and vomiting [37]. However, there is currently no COS for clinical trials that investigate the effects of interventions to control chemotherapy-induced nausea and vomiting in cancer patients. Thus, we will consider this to be a variable measurement instrument until we assess how the majority of studies included in this review are measured. In addition to CINV, other variables investigated in the selected studies will also be identified.

The secondary outcomes to be considered are the possible adverse effects from using auriculotherapy as a CINV treatment in cancer patients.

##### Setting

Primary, secondary, and tertiary health care services are the setting of the study.

#### Information sources

The search for studies will be carried out in the databases: Medical Literature Analysis and Retrieval System Online (MEDLINE) via PubMed, EMBASE (via Embase.com), Cumulative Index to Nursing and Allied Health Literature (CINAHL), Cochrane Central Register of Controlled Trials (CENTRAL), World Health Organization International Clinical Trials Registry Platform (ICTRP), ClinicalTrials.gov (CT.gov), Latin-American Literature in Health Sciences (LILACS), Cuban National Medical Information Sciences (CUMED), Spanish Bibliographic Health Science Index (IBECS), Traditional, Complimentary and Integrative Medicine (MTCI Americas) via Virtual Health Library (BVS), Web of Science, Scopus, Physiotherapy Evidence Database (PEDro), China National Knowledge Infrastructure (CNKI), and Chinese Biomedical Literature Database (CBMdisc) up until December 2018. No language restrictions will be applied to this study. For each article selected for inclusion, abstracts and full articles will be obtained. Reference lists of the included studies and systematic reviews will also be examined during the review. Furthermore, ongoing studies will be identified by means of international platforms of clinical tests of the World Health Organization and Clinical Trials, and the Brazilian Registry of Clinical Tests (ReBEC).

#### Search strategy

The search strategies will be developed by two authors with the contribution of a librarian who is experienced with databases in the health area. The research will be repeated immediately after the final analysis, in order to enable new studies to be revised, and if the inclusion criteria are met, they can be included in this review. We will use Medical Subject Heading (MeSH), keywords, and free text search terms. The key search terms are ([“auricular acupuncture” OR “auricular acupressure” OR “auricular pressing” OR “auricular needle” OR “auricular plaster” OR “auricul∗” OR “auricular acupoint∗” OR “auricular point∗” OR “otopoint∗” OR “ear point∗” OR “ear acupuncture” OR “auricular acupuncture” OR “auricular therapy”] AND [“antineoplastic agents” OR “chemotherap*”] AND [“nausea” OR “vomiting”] AND [“neoplasm*” OR “cancer*” OR “carcino*” OR “malignan*” OR “tumor*” OR “tumour*”]).

We will adapt the search strategy to the remaining databases previously mentioned. The terms will be combined by means of Boolean operators.

#### Study records

##### Data management

The search will be carried out in the aforementioned databases and then loaded into EndNote software, management software for references that allows the identified references to be organized into different electronic databases. All the results will be inserted in a single EndNote folder, and the duplicated studies will be identified and removed. After the duplicate removal, the research results will be loaded onto Covidence, a software that assists the article trials, database extraction, and cooperation among many revisors. The database extraction form will be adapted for this review based on the data extraction and evaluation model proposed by the Cochrane Handbook for Systematic Reviews of Interventions [38]. This will include assessing study characteristics such as methods, participants, interventions, and outcomes.

##### Selection process

The article selection will be carried out in two stages. The first stage will involve the title and summary reading of the search results, based on the eligibility criteria. In the second stage, the pre-selected articles will be read completely to confirm whether they meet the eligibility criteria or not. In both stages, each article will be evaluated independently by two authors. In the case of disagreement between these authors regarding the eligibility status of any study, another analysis will be carried out by another two authors, and any divergence will be settled by mutual discussion. The revisors will not be blinded for the publishing periodic, authors, or institutions during any stage of the selection process. The PRISMA flow diagram will be used to illustrate the trial and study selection process.

##### Data collection process

Two authors will extract data from the included studies independently, using the aforementioned data extraction template. All the authors will revise and discuss this form before beginning the data extraction. Furthermore, all the authors will pilot test the data extraction of a single selected study to ensure that there is a consistent interpretation of the data required for extraction. To minimize the inconsistency among reviewers, we will carry out training exercises using the data extraction form before further included study data is extracted for the review.

The data extraction form will be filled out by at least two authors for each study included in this review. One of the authors will be responsible for the cross verification of each data extraction form with the purpose of the conclusion. Any differences found in the data extracted from a study in particular will be settled by a discussion between the two authors who will collect the data, and in case of disagreement between them, the study will be evaluated by another two authors. If it is identified that there is data missing, the corresponding authors of the study will be contacted by email (a maximum of three times) to obtain the data that is missing.

#### Data items

The data extraction form will be focused on the study’s design and methods, participants, intervention details, outcome measures used, and results [39] (Table 2). The details related to the intervention will be extracted based on the Standards for Reporting Interventions in Clinical Trials of Acupuncture (STRICTA), a methodological guideline for the evaluation of clinical tests regarding acupuncture [40].

**Table 2:** Data extraction form checklist

Source	Reviewer name, review date, study title and authors, journal name, and publication date
Eligibility	Confirm eligibility for review
Introduction	Study aims and hypotheses included
Methods—participants	Setting, eligibility criteria, cancer, and treatment aspects (type of cancer, disease staging, medication used for the chemotherapy)
Methods—design and group allocation	Study design and duration, group description, sequence generation, allocation concealment, implementation, and blinding
Methods—Interventions	Intervention description and intervention details (STRICTA)
Methods—outcomes	Name and definition, time points measured, measure of CINV, and other variables investigated besides CINV
Methods—statistical analyses	Statistical analyses used
Results	Number of participants randomized/allocated per group/analyzed, details of any missing participants, baseline demographics for each group, summary data for each group at each time point, compliance with intervention, and any adverse events
Discussion/conclusion	Interpretation of results, extent of generalizability, and key conclusions

#### Risk of bias in individual studies

The risk of bias evaluation will be based on the Cochrane Collaboration Risk of Bias Tool [41]. Two authors will evaluate independently the methodological quality based on the following areas: random sequence generation, allocation concealment, participants and research team blinding, result evaluation blinding, incomplete results data, selective reports, and other biases. Considering these areas, each evaluated study will be categorized in relation to the risk of bias as low, high, and not clear risk. Furthermore, biases identified as a STRICTA methodological guideline support will be considered biases related to auriculotherapy. Any disagreements will be discussed and settled by another two authors.

#### Data synthesis and analysis

If the meta-analysis can be conducted, the R (R Core Team) meta-package will be used to analyze the data [42]. The risk reason (RR) with 95% confidence intervals (CI) will be used to estimate dichotomous data, and the average IC difference of 95% will be used for continuous data. Funnel plots and Egger’s regression test will be used to examine the possible risk of publication bias. The heterogeneity among studies will be evaluated by Cochran’s *Q* method and by Higgins’s method (*I*^2^ statistics), which describes the variation percentage among studies due to heterogeneity [43]. Values of 25%, 50%, and 75% for *I*^2^ will represent, respectively, low, average, and high heterogeneity [44]. The result will be presented using a forest plot and harvest plot, when needed [45, 46].

If data are deemed inadequate to be analyzed in a quantitative way, a systematic narrative synthesis will be undertaken to present the studies included in the review.

#### Subgroup and sensitivity analysis

Subgroup analysis will be based on possible factors that may lead to heterogeneity, such as intervention, control, ages, treatment duration, and the quality of study [47]. We will use sensitivity analysis to determine the effects of the method of auriculotherapy, methodological quality, and use of intention-to-treat analysis. If there were any studies with high attrition rates (over 50%), we would remove them from the meta-analysis to determine whether the results would be significantly different without them [48].

#### Confidence in cumulative evidence

The Grading of Recommendations, Assessment, Development and Evaluation (GRADE) classification will be used to evaluate the evidence quality [49]. This evaluation will be carried out independently by two authors. In case of disagreement between them, an agreement meeting will be carried out with a third author.

## Discussion

Due to the increasing cancer incidence in the world population and consequently the rise in the need for antineoplastic chemotherapy, it is important for both healthcare professionals and patients for there to be an effective treatment for the side effects of chemotherapy such as CINV. Auriculotherapy may be a potential alternative treatment; it is low cost and appears to be safe. However, there is a lack of evidence regarding its effectiveness in controlling the CINV in patients with cancer, as well as a protocol for the use of auriculotherapy for CINV, which also hinders the application of this intervention in clinical practice. Through this review, we hope to eliminate this knowledge gap, offering an updated and comprehensive synthesis of the existing research on this topic. In the event of limited or inconclusive evidence, this review will identify an area for further research. Therefore, we believe that this systematic review is extremely important for patients who are facing the consequences of CINV and who are keen to try alternative therapies, as well as for the healthcare professionals responsible for caring for these patients.

## Additional file

Additional file 1: PRISMA-P 2015 Checklist. (DOCX 22 kb)

## Data Availability

All search strategies and all structured data extraction forms will be shared.

## Abbreviations

CAT: Complementary and alternative therapies
CBMdisc: Chinese Biomedical Literature Database
CENTRAL: Cochrane Central Register of Controlled Trials
CINAHL: Cumulative Index to Nursing and Allied Health Literature
CINV: Chemotherapy-induced nausea and vomiting
COS: Core outcome sets
CT.gov: ClinicalTrials.gov
CNKI: China National Knowledge Infrastructure
CUMED: Centro Nacional de Informação de Ciências Médicas de Cuba
GRADE: Grading of Recommendations, Assessment, Development and Evaluation
IBECS: Índice Bibliográfico Espanhol de Ciências de Saúde
ICTRP: World Health Organization International Clinical Trials Registry Platform
LILACS: Literatura Latino-Americana em Ciências da Saúde
Medline: Medical Literature Analysis and Retrieval System Online
MeSH: Medical Subject Headings
MTCI Américas: Medicinas Tradicionais, Complementares e Integrativas
PEDro: Physiotherapy Evidence Database
PICO: Patient, Intervention, Comparator, Outcome
PRISMA: Preferred Reporting Items for Systematic Review
PRISMA-P: Preferred Reporting Items for Systematic Review and Meta-Analyses Protocol
PROSPERO: Prospective Register of Systematic Reviews
ReBEC: Registro Brasileiro de Ensaios Clínicos
STRICTA: Standards for Reporting Interventions in Clinical Trials of Acupuncture
